# Modification of Proliferation and Apoptosis in Breast Cancer Cells by Exposure of Antioxidant Nanoparticles Due to Modulation of the Cellular Redox State Induced by Doxorubicin Exposure

**DOI:** 10.3390/pharmaceutics13081251

**Published:** 2021-08-13

**Authors:** Laura Denise López-Barrera, Roberto Díaz-Torres, Joselo Ramón Martínez-Rosas, Ana María Salazar, Carlos Rosales, Patricia Ramírez-Noguera

**Affiliations:** 1 Departamento de Ciencias Biológicas, Facultad de Estudios Superiores Cuautitlán, Universidad Nacional Autónoma de México (UNAM), Ciudad Universitaria, 4510, 4513, Mexico City CP 54714, Mexico; laura.lobar@cuautitlan.unam.mx (L.D.L.-B.); mvzjoselomartinez@gmail.com (J.R.M.-R.); 2 Departamento de Ingeniería y Tecnología, Facultad de Estudios Superiores Cuautitlán, Universidad Nacional Autónoma de México (UNAM), Ciudad Universitaria, 4510, 4513, Mexico City CP 54714, Mexico; diaztorres_r@hotmail.com; 3 Departamento de Medicina Genómica y Toxicología Ambiental, Instituto de Investigaciones Biomédicas, Universidad Nacional Autónoma de México (UNAM), Ciudad Universitaria, 4510, 4513, Mexico City CP 54714, Mexico; anamsm@biomedicas.unam.mx; 4 Departamento de Inmunología, Instituto de Investigaciones Biomédicas, Universidad Nacional Autónoma de México (UNAM), Ciudad Universitaria, 4510, 4513, Mexico City CP 54714, Mexico; carosal@biomedicas.unam.mx

**Keywords:** glutathione, nanoparticles, oxidative stress, doxorubicin, breast cancer

## Abstract

In this report, we investigated whether the use of chitosan-carrying-glutathione nanoparticles (CH-GSH NPs) can modify proliferation and apoptosis, and reduce cell damage induced by doxorubicin on breast cancer cells. Doxorubicin is a widely used antineoplasic agent for the treatment of various types of cancer. However, it is also a highly toxic drug because it induces oxidative stress. Thus, the use of antioxidant molecules has been considered to reduce the toxicity of doxorubicin. CH-GSH NPs were characterized in size, zeta potential, concentration, and shape. When breast cancer cells were treated with CH-GSH nanoparticles, they were localized in the cellular cytoplasm. Combined doxorubicin exposure with nanoparticles increased intracellular GSH levels. At the same time, decreasing levels of reactive oxygen species and malondialdehyde were observed and modified antioxidant enzyme activity. Levels of the Ki67 protein were evaluated as a marker of cell proliferation and the activity of the Casp-3 protein related to cell apoptosis was measured. Our data suggests that CH-GSH NPs can modify cell proliferation by decreasing Ki67 levels, induce apoptosis by increasing caspase-3 activity, and reduce the oxidative stress induced by doxorubicin in breast cancer cells by modulating molecules associated with the cellular redox state. CH-GSH NPs could be used to reduce the toxic effects of this antineoplastic. Considering these results, CH-GSH NPs represent a novel delivery system offering new opportunities in pharmacy, material science, and biomedicine.

## 1. Introduction

Breast cancer is one of the leading health problems worldwide. Its incidence is estimated at 11.6%, placing it among the first three types of cancer diagnosed in both men and women [1]. Approximately half of the people diagnosed with breast cancer usually present recurrences even after treatment and about one-third of these patients die from the disease [2]. About 80% of breast carcinomas are positive for hormonal (progesterone and estrogen) receptors. These tumors are treated with drugs such as tamoxifen that blocks estrogen-induced cell growth. Other breast tumors (about 15%) express the human epidermal growth factor receptor (HER2). These tumors are treated with a monoclonal antibody such as trastuzumab, which is specific against HER2. The third group of breast tumors does not express hormonal receptors or HER2 and is known as triple-negative.

These tumors tend to be more aggressive, and their treatment is based on the general inhibition of cell replication on all dividing cells [2].

Doxorubicin (Dox) is a potent broad-spectrum antineoplastic agent belonging to the anthracycline family and is used to treat various cancer types including breast cancer. Its action mechanism is associated with inhibiting cell replication by binding to the enzyme topoisomerase II, causing DNA alterations, and favoring the aging of cells [3]. Unfortunately, it also induces oxidative stress that can affect both dividing and non-dividing cells. Consequently, doxorubicin can trigger undesirable side effects due to general cell toxicity. Dox stimulates the formation of free radicals (O_2_^−^, H_2_O_2_, and ^•^OH) and reactive oxygen species (ROS) through Fenton chemistry reactions during the metabolic transformation of doxorubicin to doxorubicinol. Besides, this antineoplastic can activate the NADPH oxidase and modify calcium metabolism [4]. Exposure to doxorubicin has also been reported to decrease the Ki67 protein levels associated with cell proliferation and to increase the number of apoptotic cells in a dose-dependent manner [5].

There is a growing interest in finding ways of reducing oxidative stress in tissues during doxorubicin treatment. A promising approach is the use of antioxidant molecules [6,7]. Glutathione (GSH) is one of the primary endogenous antioxidants at the cellular level and is associated with various events such as proliferation, apoptosis, and redox state regulation. It is synthesized exclusively in the cell cytoplasm and once used and in its oxidized state, it cannot be incorporated into the cell, thus it must be synthesized to maintain the levels in an optimal state [8]. Moreover, GSH is recognized as a fundamental antioxidant molecule for cellular protection from toxins, both endogenous and environmental, including several anti-cancer cytotoxic drugs [9].

Transporting GSH and other agents into cells to reduce the toxic effects of anti-cancer drugs requires the use of innovative delivery systems [10]. Nanotechnology in cancer treatment represents a novel alternative to deliver agents to cells due to the physicochemical properties of many different nanoparticles. Chitosan (CH), a natural polymer, has been used to create nanoparticles (NPs) that are ideal delivery systems. They are easy to produce, have a shallow immunogenic profile, diffuse quickly into cells, and are biodegradable and biocompatible. In addition, CH NPs can easily interact with many other molecules due to their chemical structure [11]. CH NPs have already been reported to deliver molecules that can regulate inflammation events and sensitize cancer cells to X-ray radiation [12,13]. This report explored the use of CH-GSH NPs to modify proliferation, apoptosis, and the cellular redox state through the modulation of oxidative stress induced by doxorubicin in two breast cancer cell lines.

## 2. Materials and Methods

### 2.1. Preparation and Characterization of Nanoparticles

Chitosan-carrying-glutathione nanoparticles (CH-GSH NPs) were prepared by the ionic gelation technique as previously described [14]. CH-GSH NPs were also coupled to rhodamine-123 at a concentration of 0.5 mg/mL in methanol for confocal microscopy analysis. Rhodamine-123 was added to the already formed CH-GSH NPs at 1: 4 ratio overnight. Nanoparticles were ultracentrifuged with glycerol at 27,000 rpm for 1 h and the ring formed containing the nanoparticles was resuspended in 1% acetic acid. Next, nanoparticles were characterized in concentration with a hydrodynamic diameter and zeta potential using the equipment Nanosight and Zetasizer from Malvern Panalytical, Malvern, UK Quantification of the encapsulated GSH was determined indirectly by the DTNB technique at a wavelength of 425 nm as described [15]. Finally, were observed the shape of CH-GSH NPs through transmission electron microscopy.

### 2.2. Cell Lines 

The breast cancer cell lines MCF-7 (ATCC HTB-22) and MDA MB-231 (ATCC HTB-26) were used. MCF-7 cells are positive for functional estrogen receptors and MDA MB-231 cells are negative for estrogen receptors, progesterone receptors, and the E-cadherin. Cells were cultured in a DMEM medium supplemented with 12% fetal bovine serum (FSB), antibiotic 1% (penicillin/streptomycin) at 37 °C in a 5% CO_2_ incubator. Cells were cultured in either 24-well or 6-well tissue culture plates until they were confluent before performing the various assays described next.

### 2.3. Confocal Microscopy Analysis

NPs coupled to rhodamine-123 were added to MCF-7 and MDA MB-231 cells, and two concentrations of NPs were used, namely 1.8 × 10^8^ nanoparticles/mL (equivalent to 0.08 mM GSH) and 1.4 × 10^9^ nanoparticles/mL (equivalent to 0.64 mM GSH), for 2 h. Then, cells were washed with PBS, fixed with 3% paraformaldehyde, and stained with DAPI (0.1 µg/mL). Finally, cells were observed with the confocal microscopy Zeiss LSM800. The images were taken at a 63× magnification at an λ ext 360/em 460 (DAPI) and λ ext 507/em 529 nm (Rhodamine 123).

### 2.4. Cytotoxicity

To evaluate the cytotoxic effect, cells were exposed to 5 µM of Dox for 12 h, then washed once with PBS, and were exposed for 2 h to CH NPs and CH-GSH NPs at a concentration of 1.8 × 10^8^ or 1.4 × 10^9^ NP/mL. We used the resazurin technique. This assay evaluates cells’ ability to reduce resazurin to resorufin and can be read at a wavelength of 570/600 nm [16]. The cells were placed in 24-well plates; treatments were added; cells were washed with PBS; resazurin was added at a concentration of 0.01% and was left to incubate for 30 min at 37 °C; and lastly, the lectures were made. To know the differences between the treatments with NPs, Dox, and the untreated cells, the following calculation was performed:

$$\frac{\left(A\;1\right)-\left(A\;2\right)\;treated\;cells}{\left(A\;1\right)-\left(A\;2\right)\;Untreated\;cells}\;\times \;100=\%\;\mathrm{Cell}\;\mathrm{viability}$$

where:

A1 = absorbance at 570 nm and

A2 = absorbance at 600 nm.

### 2.5. Biomarkers of Oxidative Stress

#### 2.5.1. Intracellular and Extracellular GSH Concentrations

Confluent cells cultured in 6-well plates were exposed to 5 µM Dox for 12 h. After time, the medium was removed, and cells were washed with PBS. Then, CH NPs or CH-GSH NPs were added for 2 h at a concentration of 1.8 × 10^8^ or 1.4 × 10^9^ NPs/ mL. At the end of the exposure time, the culture medium was removed and washed with PBS twice, placed in 100 µL of lysis buffer (0.1% Triton, 5 mM EDTA, 1 mM PMSF), and scraped on ice. The cell suspension was centrifuged at 13,000 rpm for 10 min at 4 ° C and the supernatant (cell lysate) was transferred to a clean tube. The amount of total protein in the cell lysates was determined according to the Bradford method [17]. The extracellular concentration of GSH was indirectly measured using the culture medium of the cells treated with nanoparticles. The concentration of intracellular and extracellular GSH was determined with the 2,2-dithiobisnitrobenzoic acid (DTNB) assay [15]. This assay is based on the reaction of GSH with DTNB, forming a yellow adduct product (GS-TNB) that can be read spectrophotometrically at a wavelength of 425 nm.

#### 2.5.2. Malondialdehyde Concentration

Malondialdehyde (MDA) concentration was determined with the thiobarbituric acid reactive species assay (TBARS) [18] with some modifications. This assay is based on the reaction of MDA with thiobarbituric acid to form a pink adduct product. The product can be read spectrophotometrically at a wavelength of 540 nm. Cells were treated with 5 µM Dox for 12 h, after which the medium was removed, and cells were washed twice with PBS. Then, CH NPs or CH-GSH NPs were added for 2 h at a concentration of 1.8 × 10^8^ or 1.4 × 10^9^ NPs/mL. At the end of the incubation time, the medium was removed, and two washes were carried out with PBS; 100 µL of lysis buffer was added; and the cells were scraped on ice. The cell suspension obtained is centrifuged at 13,000 rpm for 10 min and the supernatant obtained is separated. The cell lysate was mixed at a 1:1 ratio with 0.67% (m/v) thiobarbituric acid and incubated at 90 °C for 30 min. Finally, samples were read spectrophotometrically at a wavelength of 540 nm.

#### 2.5.3. Measurement of ROS

The detection of reactive oxygen species was performed with the dichlorofluorescein diacetate (DCFDA) assay [19] with some modifications. Cells were treated for 12 h with 5 µM Dox, underwent two washes carried out with PBS, and CH NPs and CH-GSH NPs were added at a concentration of 1.8 × 10^8^ or 1.4 × 10^9^. At the end of the exposure time, the culture medium was removed, and the cells were washed twice with PBS. The fluorogenic dye 2’,7´-DCFDA was added to the cells at 5 μM and were incubated for 15 min at 37 °C. In the presence of the reactive oxygen species and other peroxides, DCFDA is oxidized to 2´,7´-dichlorofluorescein (DCF), a fluorescent product that can be detected with a fluorometer at excitation light ʎex = 488 nm and emission light ʎem = 525 nm.

#### 2.5.4. Antioxidant Enzymes’ Activity

To estimate the activity of antioxidant enzymes, the cells were treated with Dox at a concentration of 5 µM for 12 h. After that time, two washes with PBS were carried out and CH NPs and CH-GSH NPs were added to the cells for 2 h at a concentration of 1.8 × 10^8^ and 1.4 × 10^9^ NPs/mL. Confluent cells were scraped and placed in 100 µL lysis buffer (0.1% Triton, 5 mM EDTA, 1 mM PMSF). Cells were centrifuged at 13,000 rpm for 10 min at 4 °C and cell lysate was transferred to a clean tube.

Catalase activity was estimated as previously reported [20]. Regarding the cell lysate, 100 µL was taken and 100 µL of the reaction medium containing ×100 (1%) was added, after which the cells were incubated at 37 °C for 15 min. Then, 100 µL of a 30% H_2_O_2_ solution was added. The enzyme-generated oxygen bubbles trapped by triton X-100 were visualized as foam. The height of the foam layer corresponds to the catalase activity and it is compared to a calibration curve made with known concentrations of catalase.

The activity of glutathione peroxidase GPx was estimated as previously reported [21]. The technique is based on measuring the decrease in NADPH absorbance at 340 nm absorption by a coupled reaction with glutathione reductase (GPx). The GPx uses GSH to convert H_2_O_2_ to H_2_O. As a result, the GSSG produced is regenerated by GRx with the conversion of NADPH to NADP^+^.

The activity of GRx was estimated as previously reported [21]. The cells were processed in the same way as glutathione peroxidase. Glutathione reductase activity reduces the oxidized form of glutathione, disulfide glutathione (GSSG), to reduced glutathione. Considering this reaction is coupled to NADPH’s oxidation and NADPH absorbs light at 340 nm, a decrease in absorbance reflects its oxidation.

### 2.6. Caspase-3 Activity

To evaluate the effect on apoptosis, the cells were treated with Dox at a concentration of 5 µM for 12 h; subsequently, two washes with PBS were carried out and CH NPs and CH-GSH NPs were added for 2 h at a concentration of 1.8 × 10^8^ and 1.4 × 10^9^ NPs/mL. At the end of the exposure time, the cells were washed with PBS and 100 µL of lysis buffer was added. Cells were scraped on ice. The suspension obtained was centrifuged at 13,000 rpm for 10 min. The supernatant (cell lysate) was collected to evaluate caspase-3 activity. Caspase-3 activity was measured in the cell lysate using the CaspACE assay System colorimetric kit, which consists of a colorimetric assay based on the spectrophotometric detection of p-nitroanilide chromophore at a wavelength of 405 nm [22].

### 2.7. Quantification of Ki67

To determine the effect on proliferation, the amount of antigen was measured by an ELISA test in which a color change from blue to yellow in the plate containing the antibody coupled to a chromogen is proportional to the Ki67 in the sample, which can be read at a wavelength of 450 nm [23]. The cells are treated with Dox at a concentration of 5 µM for 12 h; subsequently, two washes with PBS were carried out and CH NPs and CH-GSH NPs were added for 2 h at a concentration of 1.8 × 10^8^ and 1.4 × 10^9^ NPs/mL. At the end of the exposure time, the cells were washed with PBS, 100 µL of lysis buffer was added, the cells were scraped on ice, and the suspension obtained was centrifuged at 13,000 rpm for 10 min. The supernatant obtained is added to the microplate to determine the presence of the antigen.

### 2.8. Statistical Analysis

Three independent biological experiments were carried out in triplicates for each experiment. The results obtained were analyzed using a one-way analysis of variance (ANOVA), followed by multiple comparisons of means according to Tukey’s statistical test, considering a significant difference at *p* < 0.05. OriginLab graphing and data analysis software was used.

## 3. Results and Discussion

### 3.1. Characterization of Nanoparticles

**Figure 1:**
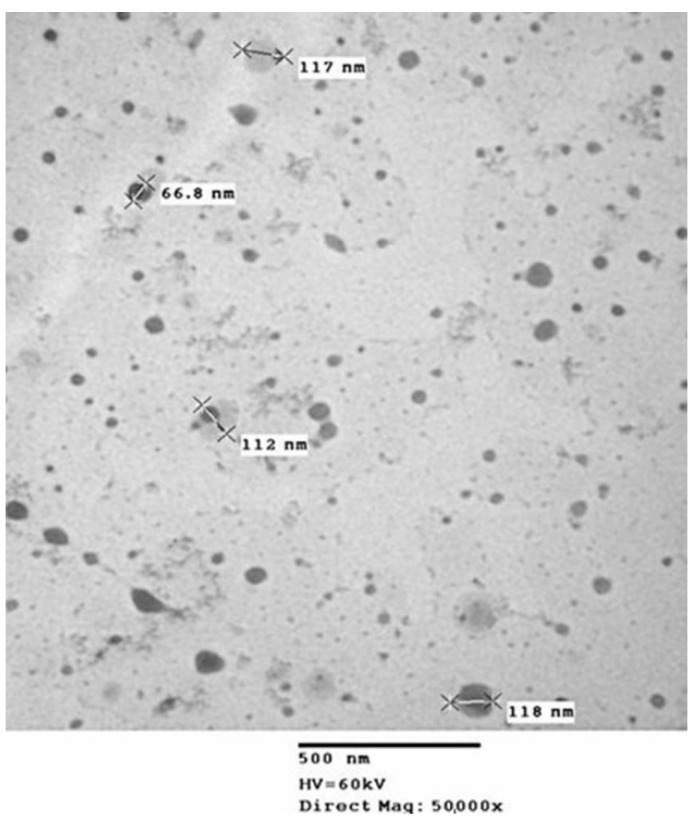
Image of transmission electron microscopy of CH-GSH NPs.

The CH-GSH NPs were prepared according to the ionic gelation method described in the experimental section. NPs were characterized by measuring their hydrodynamic diameter, polydispersion index (PDI), zeta potential, concentration, and GSH encapsulation percentage (Table 1). CH-GSH NPs labeled with rhodamine 123 had a hydrodynamic diameter between 100 and 150 nm. The particle size is an important parameter because it is assumed that most nanoparticles can be transported into the cells by endocytosis [24]. The polydispersion index indicated that both preparations of nanoparticles were homogeneous suspensions. This argument was further supported by the zeta potential which suggested that the nanoparticles remained in suspension without precipitation [25]. The percentage of GSH encapsulation was 99.23%, indicating that enough GSH was captured in the NPs. Considering GSH is very hydrophilic, it cannot enter cells unless it is trapped inside a nanocarrier. In addition, analysis of the nanoparticles’ characterization by transmission electron microscopy showed that most particles were spherical (Figure 1).

### 3.2. Chitosan-Carrying-Glutathione Nanoparticles (CH-GSH NPs) Are Localized into the Cells

**Figure 2:**
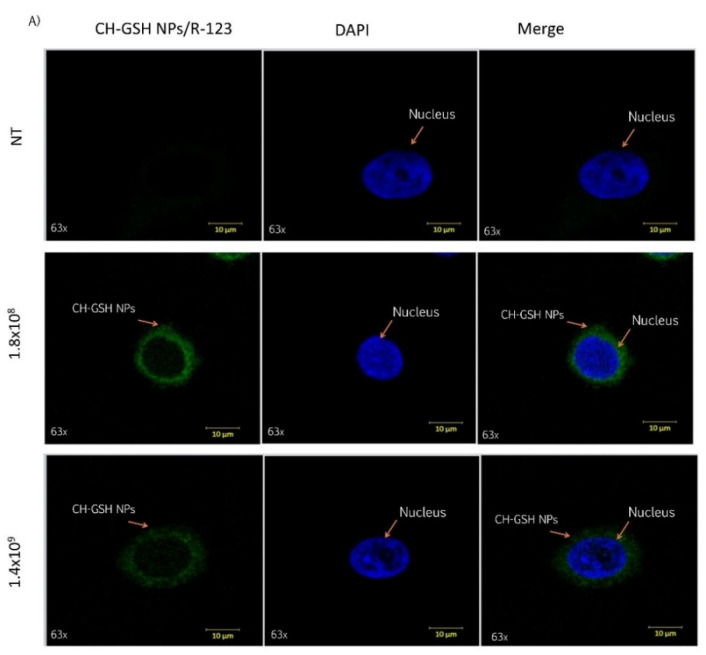

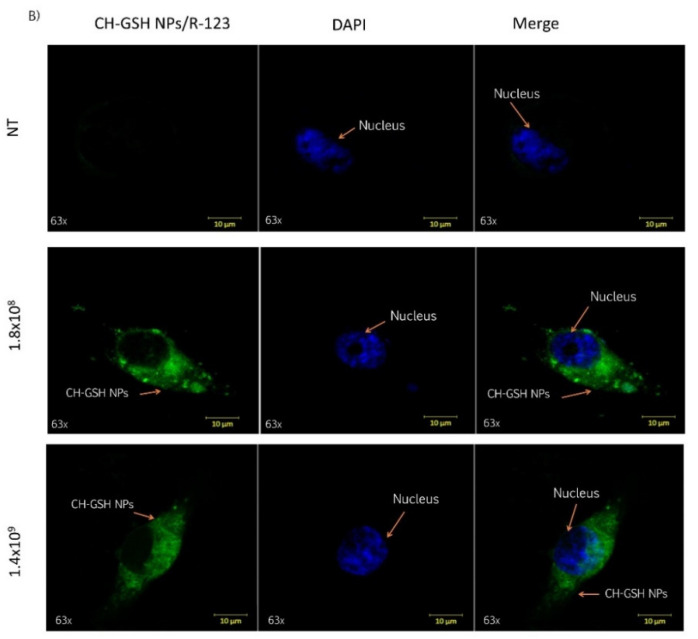
Confocal microscopy images of cells exposed to a concentration of 1.8 × 10^8^ NPs/mL and 1.4 × 10^9^ NPs/mL at the time of 2 h. Untreated cells (NT), (**A**) MCF-7 and (**B**) MDA MB-231 cells.

Cells were exposed to two different concentrations of CH-GSH NPs labeled with rhodamine-123 for 2 h and then subsequently stained with DAPI to differentiate the nucleus. Two different breast cancer cell lines readily internalized the NPs, accumulated in the cytoplasm near the nucleus’ periphery. As shown in Figure 2, CH-GSH NPs are in the cytoplasm in both cell lines. We used two different NPs concentrations; however, no significant differences were observed in the images obtained. Qualitative observations suggest a greater sensitivity in the distribution of CH-GSH NP in the cytoplasm of MDA MB-231 cells even at the lowest exposure dose compared to the MCF-7 cell line. In this case, the higher doses tested showed a minor inclusion in MCF-7 cells. These results suggest sharp differences to the nanoparticles studied.

### 3.3. Nanoparticles Do Not Reduce the Cell Viability and Do Not Alter Cytotoxicity Induced by Doxorubicin

**Figure 3:**
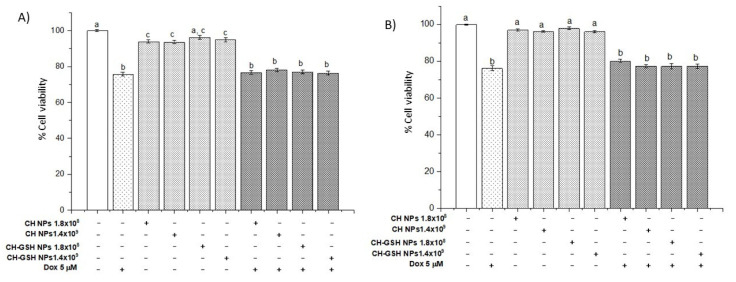
Viability of cells exposed to doxorubicin for 12 h and then 2 h with NPs. (**A**) MCF-7 and (**B**) MDA MB-231 cells. Bars with equal letters indicate no significant differences between the means (Tukey’s test, *p* < 0.05).

To demonstrate that exposure to NPs did not compromise cell viability, a resazurin assay was performed [16]. As shown in Figure 3, the exposure to 5 µM of doxorubicin and its combination with nanoparticles in the two concentrations did not show significant differences between them, suggesting that the presence of NPs does not alter the cytotoxic capacity of doxorubicin.

### 3.4. Doxorubicin Exposure with a Nanoparticle Increase the Intracellular GSH Levels

**Figure 4:**
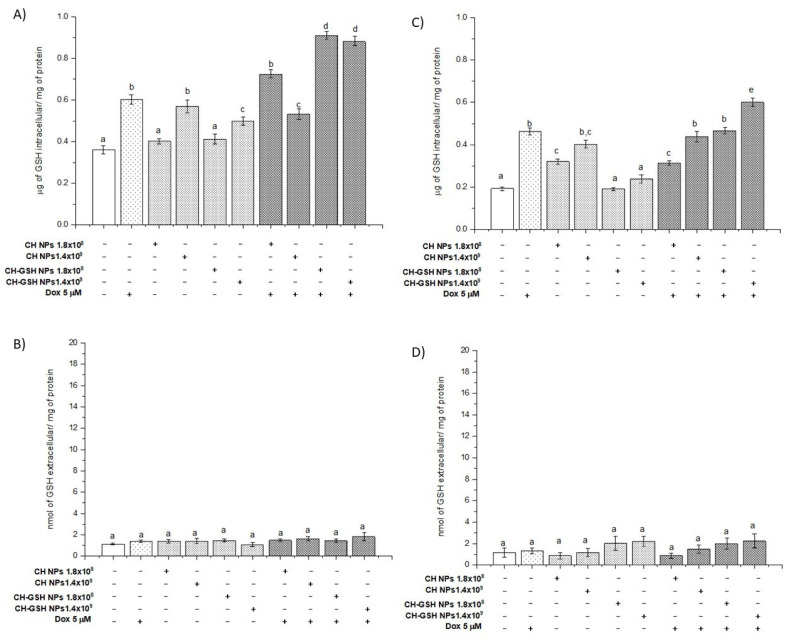
GSH intra (**A**,**C**) and extracellular (**B**,**D**) levels of cells exposed to doxorubicin for 12 h and then 2 h with CH-GSH NPs or CH-NPs. MCF-7 (**A**,**B**) and MDA MB-231 (**C**,**D**) cells. Bars with equal letters indicate no significant differences between the means (Tukey’s test, *p* < 0.05).

Total intracellular and extracellular GSH concentrations were determined in cells exposed to the NPs. Intracellular GSH concentration increased significantly compared with the untreated cells when the cells were exposed to CH-GSH NPs and CH-NPs with the highest concentration tested. The MDA MB-231 cells were the exception (Figure 4A,C). For MDA MB-231 cells, the NPs treatment did not change the intracellular concentration of GSH (Figure 4C). These results suggest differences in the susceptibility of exposed cells by having available GSH content in the NPs. The exposure only to nanoparticles in the cells does not modify the intracellular GSH and statistically GSH levels are like those obtained in the untreated cells. In combined treatments, levels may increase due to previous exposure to the stress-inducing agent doxorubicin.

Exposing cells to doxorubicin (Dox) significantly increased the intracellular GSH concentration and this concentration was higher in the combined exposure to Dox and CH-GSH NPs. This increase was more evident in MCF7 cells than in MDA MB-231 cells (Figure 4A,C). We quantified the extracellular GSH levels in the culture medium used in cells exposed to Dox. CH-GSH NPs and CH-NPs in Figure 4B,D showed no significant differences between extracellular GSH levels in any culture media for the treatments and any cell lines. This finding indicates that the NPs do not leak the GSH. The contrast between intracellular and extracellular GSH values suggests nanoparticles’ inclusion, correlates with confocal microscopy images, and suggests the bioavailability of thiol in NPs.

Interestingly, the results show an increase in the GSH concentration dose-response when MDA MB-231 cells were exposed to CH-NP. This effect was not present in the same way in the MCF-7 cells; it was only significant in the maximum concentration studied and this amount was higher than that induced when the cells were exposed to CH-GSH NPs. There is documented evidence that demonstrates the ability of CH-NP to modulate various cellular signals associated with exogenous stimuli such as radio-sensitization, antioxidation, and changes in cell unions, among many others. In addition to this, the physicochemical nature of the nanoparticles can generate cellular responses that significantly promote changes in the concentration of GSH; in turn, this promotes different effects related to enzymatic or non-enzymatic events, which finally control its cellular concentration [8,9]. NPs, as a xenobiotic agent, could exert this type of phenomenon that should be further studied.

### 3.5. Lipoperoxidation Levels Are Reduced by the Combination of Doxorubicin and NPs

**Figure 5:**
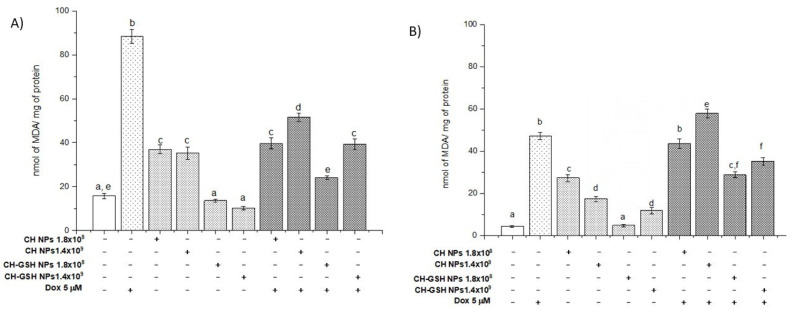
MDA levels of cells exposed to Dox for 12 h and then 2 h with CH-GSH NPs or CH-NPs. (**A**) MCF-7 and (**B**) MDA MB-231 cells. Bars with equal letters indicate no significant differences between the means (Tukey’s test, *p* < 0.05).

Malondialdehyde (MDA) is the final product of lipid oxidation. Thus, it is an indicator of cellular damage due to oxidative stress. The treatment of MCF-7 cells with Dox resulted in a marked increase in MDA concentration (Figure 5A). A similar result was observed in MDA MB-231 cells (Figure 5B). Exposure of cells to the CH-GSH NPs did not change the basal concentration of MDA in MCF7, indicating that the NPs alone did not induce oxidative stress on the cells. This effect differed in MDA MB-231 cells (Figure 5B), suggesting differential sensibility to induce MDA. The physicochemical properties of CH-NPs promote increasing levels of MDA. Combined exposure to Dox and subsequently to CH-GSH NPs resulted in a substantial reduction of MDA concentration (Figure 5). This result suggests that CH-GSH NPs have a protective antioxidant effect. Even cells exposed to CH-GSH NPs maintained MDA levels like untreated cells, while exposure to Dox significantly increased MDA levels in both cell lines (Figure 5). Therefore, these data suggest that the GSH into NPs could interact directly with ROS and free radicals or be used by antioxidant enzymes to reduce oxidative stress produced by the exposure to Dox [8].

### 3.6. Exposure to the Combination of Doxorubicin and CH-GSH NPs Reduces ROS Levels

**Figure 6:**
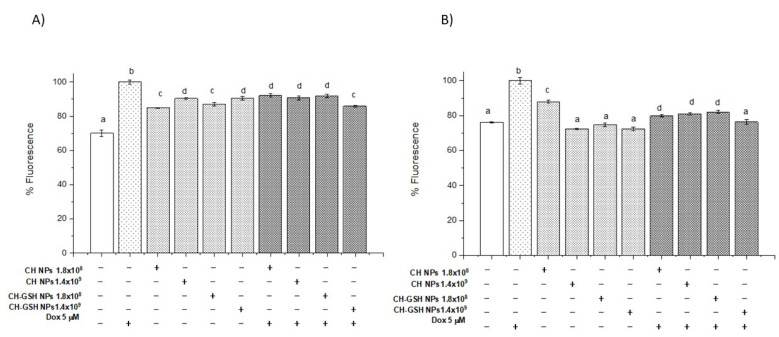
Combined exposure to Dox and CH-GSH NPs or CH-NPs reduced ROS levels. The cells were exposed to Dox for 12 h and then 2 h with CH-GSH NPs. (**A**) MCF-7 and (**B**) MDA MB-231 cells. Bars with equal letters indicate no significant differences between the means (Tukey’s test, *p* < 0.05).

Considering the NPs could modify the amount of intracellular GSH and the lipid peroxidation by reactive species decreased significantly in the combined exposures compared to Dox alone, we decided to estimate ROS using 2, 7 dichlorofluorescein diacetate (DCFDA). Figure 6A,B shows normalized results considering the untreated cells as baseline ROS levels. Cells exposed to CH-GSH NPs did not change the basal levels of ROS. As anticipated, cells exposed to Dox had a higher amount of ROS. In contrast, the combined exposure to Dox and CH-GSH NPs resulted in a marked reduction of ROS levels. This implies that the modulating effects modify the amount of ROS at the cellular level due to CH-GSH NPs indeed inducing a protective antioxidant effect in cells exposed to Dox.

Cell responses to CH-GSH NPs and CH-NP previously exposed to doxorubicin promoted a significant decrease in ROS generation compared to the amount of ROS in cells exposed to doxorubicin in both exposed cell lines. There is evidence reported concerning the CH-NP inducing antioxidant effects due to its ability to provide chelating ligands in the amino and hydroxyl groups in positions C-3 and C-2 in monomers, and effectively chelate heavy metals as Fe^2+^.

It has also been reported that these reactive groups may be responsible for the capture of some free radicals [26,27,28].

Notably, ROS levels also decreased in cells treated with doxorubicin and CH-NPs in MCF-7 cells compared with cells exposed to Dox. These effects may be related to their antioxidant capacity previously reported to CH-NPs and the cellular effects on inducing gene expression and biochemical regulation related to the modulation of the intracellular redox status.

### 3.7. Doxorubicin Decreases the Activity of Antioxidant Enzymes Induced by CH-GSH NPs but It Depends on the Cell Type

**Figure 7:**
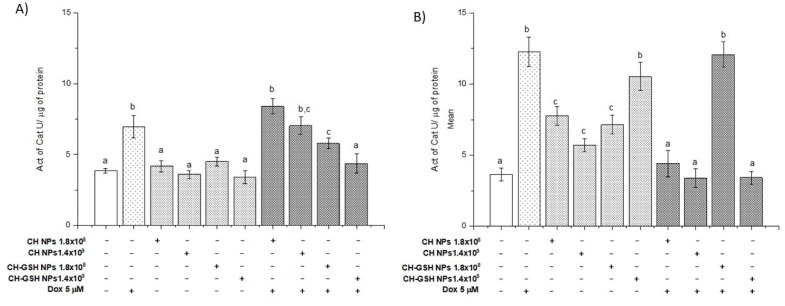
The activity of catalase. The cells were exposed to Dox for 12 h and then 2 h with CH-GSH NPs or CH-NPs. (**A**) MCF-7 and (**B**) MDA MB-231 cells. Bars with equal letters indicate no significant differences between the means (Tukey’s test, *p* < 0.05).

We quantified the specific activity of catalase. In Figure 7, CH-GSH NPs only modified MDA MB-231 cells’ activity, while in MCF-7 cells, there is no difference concerning the untreated cells. However, when cells are exposed to Dox and CH-GSH NPs, the activity is diminished compared to the Dox-induced. As previously observed, the GSH from NPs modified ROS levels, decreasing catalase activity for this reason. Catalase is the enzyme responsible for the degradation of H_2_O_2_ to H_2_O. Thus, it has a protective antioxidant effect on the cell. The catalase activity in MCF-7 cells did not change after exposing the cells to CH-GSH NPs (Figure 7A).

In contrast, the catalase activity in MDA MB-231 cells was increased by exposing the cells to NPs (Figure 7B). This variation in response reflects important metabolic differences in both cell lines. Despite this, both cell lines presented a marked increase in catalase activity after the treatment with Dox, inhibited by NPs presence.

**Figure 8:**
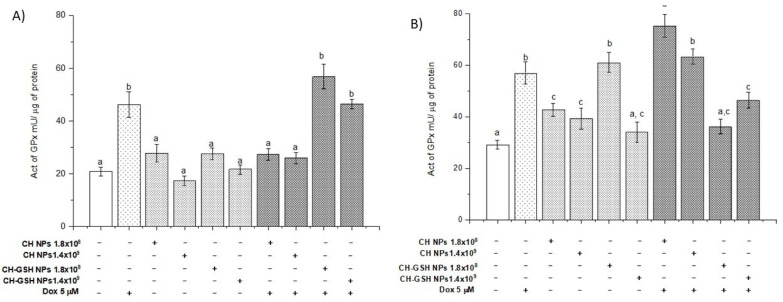
The activity of GPx. The cells were exposed to Dox for 12 h and then 2 h with CH-GSH NPs or CH-NPs. (**A**) MCF-7 and (**B**) MDA MB-231 cells. Bars with equal letters indicate significant differences between the means (Tukey’s test, *p* < 0.05).

The activity of glutathione peroxidase (GPx) (Figure 8) shows that the enzyme activity only modified MDA MB-231 cells in a concentration of 1.8 × 10^8^. When cells are exposed to Dox combined with CH-GSH NPs, the activity increases in levels like Dox-induced in the MCF-7 cells and the activity decreased in the MDA MB-231 cells. The enzyme GPx is part of the intrinsic antioxidant mechanisms by reducing peroxides with the aid of GSH as a reducing agent at the cellular level. The basal activity of GPx in MCF-7 cells did not change after exposing them to CH-GSH NPs (Figure 8A). In contrast, GPx activity in MDA MB-231 cells was increased after exposing the cells to a concentration of 1.8 × 10^8^ NPs (Figure 8B). Again, this variation in response seems to reflect important metabolic differences in both cell lines. Both cell lines presented a marked increase in GPx activity after the treatment with Dox (Figure 8), which was not inhibited by the NPs presence in MCF-7 cells (Figure 8A). In contrast, the NPs induced a significant decrease in GPx activity in MDA MB-231 cells after the treatment with Dox (Figure 8B).

**Figure 9:**
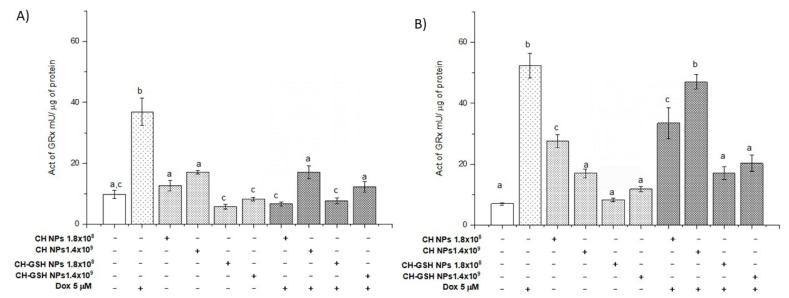
The activity of GRx. The cells were exposed to Dox for 12 h and then 2 h with CH-GSH NPs or CH-NPs. (**A**) MCF-7 and (**B**) MDA MB-231 cells. Bars with equal letters indicate no significant differences between the means (Tukey’s test, *p* < 0.05).

Glutathione (GSH) functions as a reducing agent during the elimination of peroxides by being oxidized and converted into disulfide glutathione (GSSG). Later, the enzyme glutathione reductase (GRx) uses GSSG as a substrate to regenerate GSH [21]. GRx is induced under oxidative stress. Thus, its activity is also indicative of the antioxidant state of a cell. The basal activity of GRx in MCF-7 cells and MDA MB-231 cells did not change after exposing them to CH-GSH NPs (Figure 9). Both cell lines presented a marked increase in GRx activity after the treatment with Dox, wholly blocked in the NPs presence. The result suggested that NPs induced a decrease in the ROS amount in the cell and consequently the cell did not require the activation of GRx.

### 3.8. CH-GSH NPs Induce Apoptosis by Increasing Caspase-3 Activity

**Figure 10:**
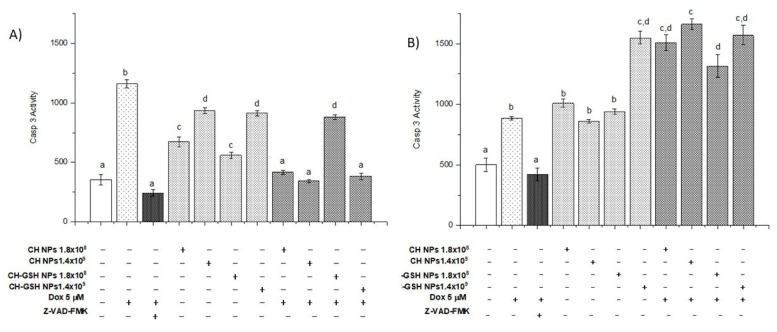
The activity of caspase-3. The cells were exposed to Dox for 12 h and then 2 h with CH-GSH NPs or CH-NPs. (**A**) MCF-7 and (**B**) MDA MB-231 cells. Bars with equal letters indicate no significant differences between the means (Tukey’s test, *p* < 0.05).

In addition to the treatments mentioned, Z-VAD-FMK, an inhibitor of caspase-3 activity, was added to demonstrate that activity is decreased when exposed to doxorubicin. In Figure 10, exposure to CH-GSH NPs increases caspase-3 activity, more evident in MDA-MB-231 cells than in MCF-7 cells. When exposure to doxorubicin followed by CH-GSH NPs occurs, it can be observed that in MCF-7 cells, the activity seems to decrease (Figure 10A). In contrast, in MDA-MB-231 cells, the activity increases concerning that induction by doxorubicin (Figure 10B). Wójcik et al., 2015, and Daga et al., 2016, suggested that the combined exposure of GSH and doxorubicin induced cell-signaling, related to the increase of apoptosis effects [29,30].

In this work, the effects associated with apoptosis were not exclusive to the chemotherapeutic agent and glutathione; some authors report events in which chitosan has an essential role in apoptosis. In studies carried out on bladder tumor cells, Hasegawa et al. showed that chitosan could induce apoptosis through the activation of caspase-3. However, the mechanism of action through which it carries out this activation is unknown [31]. Lee et al., 2011, showed that a derivative of chitosan, diethylaminoethyl chitosan, could induce apoptosis in Hela cells through the regulation of enzymes (caspase-3, -8, and -9); p53 and BAX expression. Modifying the expression of BCL2 proteins. This would generate a disruption of the mitochondrial membrane and an oxidation–reduction imbalance [32].

Wimardhani et al., 2014, reported that the exposure of Ca9-22 cells with chitosan derivatives induced the appearance of early apoptotic cells, increased caspase-3 activity, and the arrest of G1/S of the cell cycle, suggesting that chitosan could be used as a natural anti-cancer agent [33]. Therefore, the caspase-3 activity increase could be due to the exposure to doxorubicin, GSH of NPs, and chitosan.

### 3.9. CH-GSH NPs Impair Cell Proliferation by Decreasing Ki67 Levels

**Figure 11:**
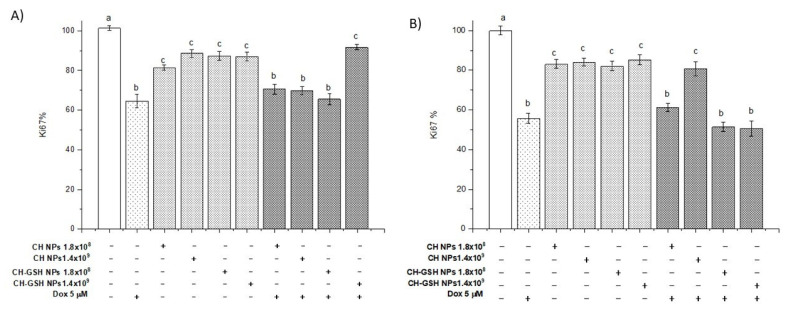
Ki67 levels. The cells were exposed to Dox for 12 h and then 2 h with CH-GSH NPs. (**A**) MCF-7 and (**B**) MDA MB-231 cells. Bars with equal letters indicate no significant differences between the means (Tukey’s test, *p* < 0.05).

Ki67 levels were measured as a molecular marker of cell proliferation. The Ki67 antigen has a specific expression in the M phase of the cell cycle; it is commonly visualized with the MIB1 antibody [34]. Our data showed that the CH-GSH NPs in both concentrations significantly decreased the percentage of Ki-67 concerning the NT cells in both cell lines (Figure 11). It has been reported that GSH levels can regulate the activity of genes associated with proliferation, differentiation, and apoptosis, and a high level of GSH is essential for normal cellular functions, signal translation, and protection against certain carcinogens [35]. When cells are exposed to both doxorubicin and CH-GSH NPs, the levels remain like those obtained with cells exposed only to doxorubicin. The above suggests that NPs could not affect doxorubicin-induced proliferation but could modify the redox state as observed when ROS levels were estimated.

Furthermore, there is evidence that the decrease in Ki67 levels is related to some chitosan derivatives, which modify various cell-signaling pathways directly or indirectly related to cell proliferation and apoptosis. Chitosan can inhibit pro-inflammatory molecules such as TNF-α, blocking the activation of NFkB and reducing the expression of genes that protect and induce cell proliferation [36]. In addition, it can activate the transcription factor Nrf2 through the PI3/Akt pathway. Rojo de la Vega et al. reported that a decrease in the expression of Nrf2 promotes the activation of CDk2 and CDk4 inhibitor p21 [37]. Another study suggests that some cells treated with chitosan derivatives can increase the expression of TGF-β that activates an intracellular-signaling cascade associated with Smad proteins and thus favors the transcription of genes associated with cell cycle inhibition [38]. MCF-7 cells showed increased sensitivity to NPs’ exposure compared to MDA MB-231 cells in the various tests performed. This finding may be due to its metabolism. It has been reported that Dox modifies the metabolism of both cell lines by affecting more the metabolic profile of MDA-MB-231 cells than of MCF-7 cells, showing changes in ketonic bodies, glycolysis, and energetic and lipid metabolism [16,39]. MDA MB-231 cells have a higher consumption of glucose and upregulated redox pathways than MCF-7 [40].

Conversely, the observed effect may be due to, as some authors mentioned, the fact that combined exposure of NPs with various agents enhances cell sensibilization. Zalbielca et al., 2017, and Willmann et al., 2015, showed that combined exposure of NPs of Dox/GSH has higher cytotoxic effects than free Dox in feline fibrosarcoma cell lines. In a study in MCF-7 cells with metallic NPs and radiation, there was a higher effect on the combination exposure, wherein NPs act as nano-sensitizers [6,41]. Uma et al., 2016, suggested that gold NPs act as sensitizing agents in MDA-MB-231 and MCF-7 cells, modifying cell-cycle effects, viability, and DNA damage [42]. Alvandifar et al. used a combined exposition of PLGA and verapamil NPs to improve this chemotherapeutic effectiveness and decrease the dose to have a higher effect [43]. These results suggest that the combined exposure of nanoparticles and doxorubicin may decrease the resistance of MDA MB-231 cells to these types of drugs.

## 4. Conclusions

The results obtained in this research suggest that CH-GSH NPs modify Ki67 levels and alter the apoptosis by increasing caspase-3 activity and the cellular redox state, reducing the oxidative stress generated by doxorubicin exposure. It has been documented that GSH may play a dual role in tumor progression or cancer cell death concerning cancer. The above is probably due to the fine biochemical regulation in its synthesis and the relationship of its oxidation–reduction modulating effects, as well as other enzymatic and non-enzymatic mechanisms. The effects of doxorubicin are variable and include the ability to induce resistance to chemotherapy; modify the initiation and progression of cancer; activate cell-signaling pathways related to stressful microenvironments; promote apoptosis in tumor cells; and have the radio-sensitization be induced by this antioxidant. We observed higher sensitivity of MCF-7 cells to CH-GSH NPs than MDA MB-231 cells; this may be due to each cells’ genotypic characteristics.

As a GSH delivery entity, CH-NPs attract attention. The results of this work show their capacity to diffuse quickly into cells and exert significant effects to modulate the oxidative stress induced by doxorubicin in breast cancer cells. Considering this, CH-GSH NPs must be studied as a potential designed delivery system that offers a new biomaterial with biomedical opportunities to study the molecular, biochemical, and biological mechanisms related to the cellular redox status. This information will be useful to design better therapies based on antioxidant nanoparticles.

## Figures and Tables

**Table 1:** Characterization results.

Nanoparticles	Hydrodynamic Diameter (nm) ± SD	Polydispersion Index (PDI)	Z Potential (mV) ± SD	Amount of NP (NPs/mL)	Encapsulation of GSH (%)
CH-GSH NPs	147.1 ± 75.40	0.246	15.2 ± 3.10	3.718 × 10^10^	99.23
CH NPs	126.7 ± 57.57	0.276	18.7 ± 2.04	3.718 × 10^10^	-
CH-GSH NPs R-123	129.8 ± 55.01	0.264	23.2 ± 1.12	5.343 × 10^10^	99.23

## Data Availability

The data presented in this study are available on request for the corresponding author.

## References

[1] Bray F., Ferlay J., Soerjomataram I., Siegel R.L., Torre L.A., Jemal A. (2018). Global Cancer Statistics 2018: GLOBOCAN Estimates of Incidence and Mortality Worldwide for 36 Cancers in 185 Countries. CA Cancer J. Clin..

[2] Pardee J.D. (2008). Understanding Breast Cancer. Cell Biology and Therapy—A Visual Approach.

[3] Renu K., Abilash V.G., Tirupathi Pichiah P.B., Arunachalam S. (2018). Molecular Mechanism of Doxorubicin-Induced Cardiomyopathy–An Update. Eur. J. Pharmacol..

[4] Chegaev K., Riganti C., Rolando B., Lazzarato L., Gazzano E., Guglielmo S., Ghigo D., Fruttero R., Gasco A. (2013). Doxorubicin-Antioxidant Co-Drugs. Bioorg. Med. Chem. Lett..

[5] Czeczuga-Semeniuk E., Anchim T. (2004). The Effect of Doxorubicin and Retinoids on Proliferation, Necrosis and Apoptosis in MCF-7 Breast Cancer Cells. Folia Histochem. Cytobiol..

[6] Zabielska-Koczywąs K., Dolka I., Król M., Żbikowski A., Lewandowski W., Mieczkowski J., Wójcik M., Lechowski R. (2017). Doxorubicin Conjugated to Glutathione Stabilized Gold Nanoparticles (Au-GSH-Dox) as an Effective Therapeutic Agent for Feline Injection-Site Sarcomas—Chick Embryo Chorioallantoic Membrane Study. Molecules.

[7] Songbo M., Lang H., Xinyong C., Bin X., Ping Z., Liang S. (2019). Oxidative Stress Injury in Doxorubicin-Induced Cardiotoxicity. Toxicol. Lett..

[8] Kalinina E.V., Chernov N.N., Novichkova M.D. (2014). Role of Glutathione, Glutathione Transferase, and Glutaredoxin in Regulation of Redox-Dependent Processes. Biochem. Mosc..

[9] Hui Wu J.H., Batist G. (2013). Glutathione and Glutathione Analogues; Therapeutic Potentials. Biochim. Biophys. Acta BBA-Gen. Subj..

[10] Raj S., Khurana S., Choudhari R., Kesari K.K., Kamal M.A., Garg N., Ruokolainen J., Das B.C., Kumar D. (2019). Specific Targeting Cancer Cells with Nanoparticles and Drug Delivery in Cancer Therapy. Semin. Cancer Biol..

[11] Rahmani S., Hakimi S., Esmaeily A., Samadi F.Y., Mortazavian E., Nazari M., Mohammadi Z., Tehrani N.R., Tehrani M.R. (2019). Novel Chitosan Based Nanoparticles as Gene Delivery Systems to Cancerous and Noncancerous Cells. Int. J. Pharm..

[12] Ma L., Shen C., Gao L., Li D., Shang Y., Yin K., Zhao D., Cheng W., Quan D. (2016). Anti-Inflammatory Activity of Chitosan Nanoparticles Carrying NF-ΚB/P65 Antisense Oligonucleotide in RAW264.7 Macrophage Stimulated by Lipopolysaccharide. Colloids Surf. B Biointerfaces.

[13] Piña Olmos S., Díaz Torres R., Elbakrawy E., Hughes L., Mckenna J., Hill M.A., Kadhim M., Ramírez Noguera P., Bolanos-Garcia V.M. (2019). Combinatorial Use of Chitosan Nanoparticles, Reversine, and Ionising Radiation on Breast Cancer Cells Associated with Mitosis Deregulation. Biomolecules.

[14] López-Barrera L.D., Díaz-Torres R., López-Macay A., López-Reyes A.G., Pina Olmos S., Ramírez-Noguera P. (2019). Oxidative Stress Modulation Induced by Chitosan-Glutathione Nanoparticles in Chondrocytes. Pharmazie.

[15] Hu M.L. (1994). Measurement of Protein Thiol Groups and Glutathione in Plasma. Methods Enzymol..

[16] Präbst K., Engelhardt H., Ringgeler S. (2017). Basic Colorimetric Proliferation Assays: MTT, WST, and Resazurin. Colorimetric Proliferation Assays Methods. Mol. Biol..

[17] Bradford M.M. (1976). A Rapid and Sensitive Method for the Quantitation of Microgram Quantities of Protein Utilizing the Principle of Protein-Dye Binding. Anal. Biochem..

[18] Ohkawa H., Ohishi N., Yagi K. (1979). Assay for Lipid Peroxides in Animal Tissues by Thiobarbituric Acid Reaction. Anal. Biochem..

[19] Wang H., Joseph J.A. (1999). Quantifying cellular oxidative stress by dichlorofluorescein assay using microplate reader. Free Radic. Biol. Med..

[20] Iwase T., Tajima A., Sugimoto S., Okuda K., Hironaka I., Kamata Y., Takada K., Mizunoe Y. (2013). A Simple Assay for Measuring Catalase Activity: A Visual Approach. Sci. Rep..

[21] Esworthy R.S., Chu F.F., Doroshow J.H. (1999). Analysis of Glutathione-Related Enzymes. Curr. Protoc. Toxicol..

[22] Promega (2018). CaspACE TM Assay System, CaspACE TM Assay System, Colorimetric.

[23] CUSABIO (2018). Human Antigen KI-67 (Ki-67). ELISA Kit.

[24] Monopoli M.P., Pitek A.S., Lynch L., Dawson D.A. (2013). Formation and Characterization of the Nanoparticle–Protein Corona. Nanomater. Interfaces Biol. Methods Protoc..

[25] Hans M.L., Lowman A.M. (2002). Biodegradable Nanoparticles for Drug Delivery and Targeting. Curr. Opin. Solid State Mater. Sci..

[26] Chang S.H., Wu W.C.H., Tsai G.J. (2018). Effects of chitosan molecular weight on its antioxidant and antimutagenic properties. Carbohydr. Polym..

[27] Choi H., Nam J.-P., Nah J.-W. (2016). Application of chitosan and chitosan derivatives as biomaterials. J. Ind. Eng. Chem..

[28] Kim K.W., Thomas R.L. (2006). Antioxidative activity of chitosans with varying molecular weights. Food Chem..

[29] Wójcik M., Lewandowski W., Król M., Pawłowski K., Mieczkowski J., Lechowski R., Zabielska K. (2015). Enhancing antitumor efficacy of doxorubicin by non-covalent conjugation to gold nanoparticles-In vitro studies on Feline fibrosarcoma cell lines. PLoS ONE.

[30] Daga M., Ulllio C., Argenziano M., Dianzani C., Cavalli R., Trotta F., Barrera G. (2016). GSH-targeted nanosponges increase doxorubicin-induced toxicity “in vitro” and “in vivo” in cancer cells with high antioxidant defenses. Free. Radic. Biol. Med..

[31] Hasegawa M., Yagi K., Iwakawa S., Hirai M. (2001). Chitosan induces apoptosis via caspase-3 activation in bladder tumor cells. Jpn. J. Cancer Res. Gann.

[32] Lee S., Ryu B., Je J., Kim S. (2011). Diethylaminoethyl chitosan induces apoptosis in HeLa cells via activation of caspase-3 and p53 expression. Carbohydr. Polym..

[33] Wimardhani Y.S., Suniarti D.F., Freisleben H.J., Wanandi S.I., Siregar N.C., Ikeda M.A. (2014). Chitosan exerts anti-cancer activity through induction of apoptosis and cell cycle arrest in oral cancer cells. J. Oral Sci..

[34] Mohamed H., Samy N., Afify M., Abd N., Maksoud E. (2008). Ki-67 as a potential biomarker in patients with breast cancer. J. Genet. Eng. Biotechnol..

[35] Abdalla M.Y. (2011). Glutathione as Potential Target for Cancer Therapy; More or Less is Good?. Jordan J. Biol. Sci..

[36] Kadry M.O., Abdel-Megeed R.M., El-Meliegy E., Abdel-Hamid A.H.Z. (2018). Crosstalk between GSK-3, c-Fos, NFκB and TNF-α signaling pathways play an ambitious role in Chitosan Nanoparticles Cancer Therapy. Toxicol. Rep..

[37] Rojo de la Vega M., Chapman E., Zhang D.D. (2018). NRF2 and the Hallmarks of Cancer. Cancer Cell.

[38] Farhadihosseinabadi B., Zarebkohan A., Eftekhary M., Heiat M., Moosazadeh Moghaddam M., Gholipourmalekabadi M. (2019). Crosstalk between chitosan and cell signaling pathways. Cell. Mol. LifeSci..

[39] Maria R.M., Altei W.F., Selistre-de-Araujo H.S., Colnago L.A. (2017). Effects of Doxorubicin, Cisplatin, and Tamoxifen on the Metabolic Profile of Human Breast Cancer MCF-7 Cells As Determined by 1 H High-Resolution Magic Angle Spinning Nuclear Magnetic Resonance. Biochemistry.

[40] Maria R.M., Altei W.F., Selistre-de-Araujo H.S., Colnago L.A. (2017). Impact of chemotherapy on metabolic reprogramming: Characterization of the metabolic profile of breast cancer MDA-MB-231 cells using 1 H HR-MAS NMR spectroscopy. J. Pharm. Biomed. Anal..

[41] Willmann L., Schlimpert M., Halbach S., Erbes T., Stickeler E., Kammerer B. (2015). Metabolic profiling of breast cancer: Differences in central metabolism between subtypes of breast cancer cell lines. Chromatogr. B.

[42] Uma S., Govindaraju K., Ganesh K., Prabhu D., Arulvasu C., Karthick V., Changmai N. (2016). Anti-proliferative effect of biogenic gold nanoparticles against breast cancer cell lines (MDA-MB-231 & MCF-7). Appl. Surf. Sci..

[43] Alvandifar F., Ghaffari B., Goodarzi N., Ravari N.S., Karami F., Amini M., Souri E., Khoshayand M.R., Esfandyari-Manesh M., Jafari R.M. (2018). Dual Drug Delivery System of PLGA Nanoparticles to Reverse Drug Resistance by Altering BAX/Bcl-2. J. Drug Deliv. Sci. Technol..

